# Ion mobility spectrometry combined with multivariate statistical analysis: revealing the effects of a drug candidate for Alzheimer’s disease on Aβ1-40 peptide early assembly

**DOI:** 10.1007/s00216-019-02030-7

**Published:** 2019-08-12

**Authors:** Serena Lazzaro, Nina Ogrinc, Lieke Lamont, Graziella Vecchio, Giuseppe Pappalardo, Ron M. A. Heeren

**Affiliations:** 1 Institute of Biostructures and Bioimaging (IBB), National Research Council, Via Paolo Gaifami N.18, 95126 Catania, Italy; 2 The Maastricht Multimodal Molecular Imaging institute M4I- Division of Imaging Mass Spectrometry, Maastricht University, Minderbroedersberg 4-6, 6211 LK Maastricht, The Netherlands; 3 Department of Chemical Sciences, Catania University, Viale Andrea Doria, 6, 95125 Catania, Italy

**Keywords:** Alzheimer’s disease (AD), Amyloid β-peptide oligomers, Electrospray ionization-ion mobility-mass spectrometry (ESI-IM-MS), Multivariate statistical analysis (MVA)

## Abstract

Inhibition of the initial stages of amyloid-β peptide self-assembly is a key approach in drug development for Alzheimer’s disease, in which soluble and highly neurotoxic low molecular weight oligomers are produced and aggregate in the brain over time. Here we report a high-throughput method based on ion mobility mass spectrometry and multivariate statistical analysis to rapidly select statistically significant early-stage species of amyloid-β1-40 whose formation is inhibited by a candidate theranostic agent. Using this method, we have confirmed the inhibition of a Zn-porphyrin-peptide conjugate in the early self-assembly of Aβ40 peptide. The MS/MS fragmentation patterns of the species detected in the samples containing the Zn-porphyrin-peptide conjugate suggested a porphyrin-catalyzed oxidation at Met-35(O) of Aβ40. We introduce ion mobility MS combined with multivariate statistics as a systematic approach to perform data analytics in drug discovery/amyloid research that aims at the evaluation of the inhibitory effect on the Aβ early assembly in vitro models at very low concentration levels of Aβ peptides.

**Electronic supplementary material:**

The online version of this article (10.1007/s00216-019-02030-7) contains supplementary material, which is available to authorized users.

## Introduction

**Fig. 1:**
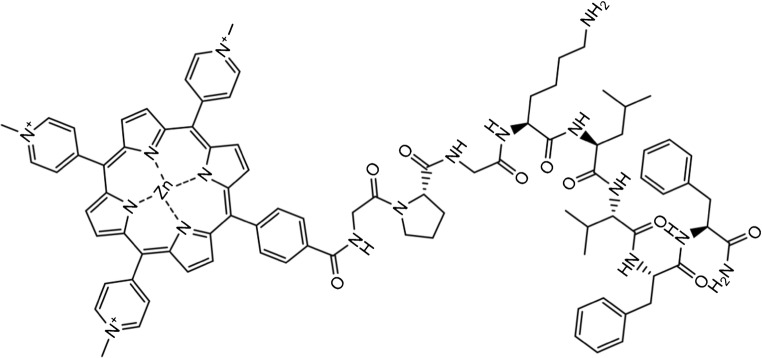
Zinc-porphyrin conjugated with the central hydrophobic motif (KLVFF) of the Aβ peptide (abbr. Zn-Porph)

Alzheimer’s disease (AD) is a neurodegenerative disorder, generally associated with the accumulation of misfolded amyloid-β (Aβ) peptides. The World Health Organization (WHO) and Alzheimer’s Disease International estimate the number of people affected by AD alone will reach 81 million worldwide by 2040, leading to a costly burden of disease [1]. Although substantial progress has been made in understanding AD, the failure of symptomatic treatments for clinically diagnosed AD in phase III clinical trials indicates that our understanding of this disease is still incomplete. The search for an effective and safe drug therefore continues. Despite the continuous debate about the amyloid hypothesis, experimental and clinical evidence support the concept that proteolytic cleavage of APP [2, 3], leaving behind soluble peptides, primarily Aβ40 and Aβ42, is a very central factor to the development of AD. Interestingly, Aβ40 is ten times more prevalent in the brain than Aβ42 but less fibrillogenic [4–9]. In addition, controversy still exists as to which of the two forms is toxic to neurons. While the Aβ monomer form remains benign [10], evidence indicates that pre-fibrillar soluble assemblies of both Aβ40 and Aβ42 peptides are the actual initiators of AD pathogenesis causing neuronal dysfunction and memory impairment [11–13]. These peptides are low molecular weight oligomers (LMWs) and not, as expected, mature end-stage amyloid fibrils. Reducing the prevalence of LMWs, intermediates with suitable inhibitors of the early-stage aggregation of Aβ peptides might decrease neuronal toxicity [14–21]. A valid therapeutic strategy proposes the use of short peptides, which recognize Aβ’s aggregation-prone amino acid sequences, as the key disruptors of Aβ’s self-assembly. It is known that the penta-peptide KLVFF, which is homologous to the Aβ(16–20) region, strongly interacts with the full-length Aβ peptide to prevent fibril formation [22, 23]. In our previous work [24], we reported the design, synthesis, and anti-fibrillogenic activity of a novel peptide conjugate (Fig. 1). This conjugate consists of a fluorescent zinc-containing porphyrin macrocycle, which is linked through a GPG peptide spacer to the KLVFF sequence of Aβ peptides (Zn-Porph).

Matrix-assisted laser desorption ionization-time of flight-mass spectrometry (MALDI-ToF-MS) experiments indicated that Zn-Porph interacts with monomeric Aβ42 in a 1:1 molar ratio [24]. Yet, Thioflavin-T (ThT) kinetics and circular dichroism (CD) data showed that Zn-Porph prevented the conformational transition of Aβ42 to a β-sheet structure. Based on these results, we hypothesize an interaction mechanism involving the zinc ion and the KLVFF peptide of the porphyrin-peptide conjugate as recognition sites of the histidine residues and hydrophobic region of Aβ42, respectively. In continuation of our studies, we would like to further elucidate whether the Zn-Porph also inhibits the Aβ early assembly. Specifically, to survey the Zn-Porph’s in vitro binding and inhibitory effects on LMWs of Aβ40 peptide. In this regard, recent studies have shown that electrospray ionization ion mobility mass spectrometry (ESI-IM-MS) is a promising analytical tool to investigate the size and conformational distribution of the Aβ early-stage LMWs in vitro models [25–36]. In respect to the screening of inhibitors [37], only a few IM-MS publications have attempted to identify small inhibitors of the initial assembly of Aβ40 at very low concentration levels [38–41]. In these studies, small molecules were added at different ratios to solutions of synthetic or recombinant Aβ40 at concentrations ranging from 10 to 32 μM. Herein, we investigated the in vitro efficacy of the Zn-Porph as the inhibitor of the early-stage assembly of synthetic Aβ40 at 5 μM and 20 μM. We combined IM-MS with multivariate statistical analysis (MVA) [42] to compare the IM-MS profiles of multiple samples [43] and to reveal a subset of statistically significant early-stage species of Aβ40 whose formation was inhibited in the presence of the Zn-Porph. From a chemometric standpoint, statistical feature selection involves discriminant techniques (supervised models). The main difference compared with the unsupervised models (PCA) is that supervised models use a priori knowledge about the class to which a specific sample belongs. Geometrically, this is the same as identifying regions in the hyperspace of the variables corresponding to the different classes [44]. Supervised models are designed to build an algorithm between a set of descriptive variables (e.g., drift time_ m/z pairs with corresponding ion intensity value in the IM-MS spectra) and the membership to a defined class of samples [45]. As a modification of the PLS algorithm, in the OPLS-DA model, the systematic variations in X are separated into two parts: one linear and one orthogonal to Y. Hence, the OPLS-DA model comprises two blocks of model variations: (1) the Y-predictive block, which represents the inter-classes variation, and (2) the Y-orthogonal block which constitutes the intra-class variation [46, 47]. The latter augments classification performance in cases where the individual classes exhibit divergence in within-class variation. This facilitates the interpretation of the model variations. In another words, OPLS-DA is an excellent tool to find “What’s the difference” between sample classes, such as between in vitro models not containing and containing a drug candidate. In case of 2-class models, indeed, the OPLS-DA S-plot helps to quickly select reliable features (drift time_ m/z pairs), which capture the bulk of the ion intensity variation between the control group (e.g., samples not containing the inhibitor) and treated group (e.g., samples containing the inhibitor). The S-plot combines the information from a traditional loading plot (PLS or OPLS) and the confidence limits column plot (plot XVariance, XVar) resulting in an easier filtering out of low confidence limit features. The plot visualizes variable according to their contribution to the inter-classes separation, based on the covariance parameter magnitude, (p[1]), and to their reliability, based on the correlation parameter value, (p(corr)[1]). Both of these two parameters have a theoretical minimum of − 1 and maximum of + 1. The selection of meaningful discriminative features needs, therefore, a combination of variable contribution (covariance, p[1]) and variable confidence (correlation, p(corr)[1]) values, which is the purpose of the S-plot. The selected features can be further ranked by the variable of importance (VIP) plot [48]. The plot ranks the overall contribution of each variable to the model taking into account both p(corr)[1] and p[1] values. The variables with VIP value greaten than 1.0 can be selected as top “reliable ions with highest discriminatory capacity.” In this study, a stringent threshold confidence interval was employed to select, among all the Aβ40 early-stage species detected in sample classes not containing the inhibitor, the meaningful ATD peaks (with low intra-class ion intensity variability) whose intensity was significantly affected by the inhibitor in the sample classes containing Zn­Porph.

## Material and methods

### Sample preparation

Samples were prepared from independent solutions of synthetic Aβ40 peptide (purity > 95%) purchased from GenScript. Eight units of 1.0 mg of solid Aβ40 were dissolved in pre-chilled 1,1,1,3,3,3-hexafluoro-2-propanol (HFIP) (Merck) to obtain a peptide concentration of 0.5 mM. The Aβ40 solution was sonicated for 5 min at room temperature (RT), the tube was chilled on ice for 1 min. The Aβ40 solution was split into aliquots in siliconized tubes. From each aliquot, the HFIP was removed under the fume hood overnight and all traces evaporated using nitrogen. The day before the MS analysis, HFIP-treated Aβ40 films were re-dissolved in dimethyl sulfoxide (DMSO, max 0.025% water, Merck). Each solution was sonicated for 5 min and subsequently incubated for 24 h at 25 °C. Prior to MS analysis, the solutions were diluted into 10 mM ammonium acetate buffer (CH3COONH4, Aldrich), pH 6.9 (in which DMSO constitutes the 1% v/v of the final volume) to a final peptide concentration of 5 and 20 μM. All samples were subsequently centrifuged at 13,000*g* for 10 min at 4 °C. The supernatant solution was stored on ice for 5 min before injection. Another set of samples at 20 μM was also incubated at 37 °C for 2 h before storing them on ice prior to ESI-IM-MS analysis. Zn-Porph (previously dissolved in CH3COONH4, 10 mM, pH 6.9) was added to monomeric Aβ40 in DMSO (as prepared above) in a 1:1 Aβ40: Zn-Porph molar ratio to study the effect on Aβ40 assembly. Summarizing, three sample sets were investigated: at 5 μM, 20 μM, and at 20 μM incubated at 37 °C for 2 h prior to injection. The 16 samples of each sample set were grouped into 2 sample classes identified as “Aβ40” (eight samples) and “Aβ40 plus Porph” (eight samples) to compare the ESI-IM-MS profiles of LMWs of Aβ40 peptide in the presence and absence of equimolar amounts of Zn-Porph. Aβ40 peptide solutions at 100 μM were used to optimize the IM-MS settings in both resolution and sensitivity mode.

### MS method and instrumentation

Direct infusion experiments were performed on a Synapt G2-Si instrument (Waters Corp., Milford, MA). Measurements were performed at a 7 μL/min injection flow rate for 5 min. Data were acquired in full scan mode using a mass range of *m/z* 800–3000 at 1 scan/sec. ESI was operated in the positive ion mode with a capillary voltage of 2.8 kV and sample cone voltage of 38 V. The source and desolvation temperatures were set at 80 and 40 °C, respectively. Nitrogen was used as a cone gas with the flow rate of 38 L/h and as desolvation gas with a flow rate of 650 L/h. The mobility T-wave cell was operated at a pressure of 3.19 mbar of nitrogen, with a wave velocity of 650 m/s and amplitude of 39 V. MS/MS spectra were acquired by CID fragmentation in the TRAP cell using collision energy of 70 V after precursor ion selection at LM resolution 6.5. Peak assignments were performed using their ^13^C isotope distributions of the species separated in the IM dimension with the MS operating in resolution mode.

### Data processing and multivariate statistical analysis

Data acquisition was carried out with MassLynx (V4.1) and DriftScope (V2.8) software. The total arrival time distribution (ATD) files classified as “Aβ40” and “Aβ40 plus Porph” were thus exported from DriftScope (V2.8) to Progenesis QI (64-bit, Nonlinear Dynamics). The Progenesis QI data analysis software is a small molecule discovery tool predominantly used to identify the significantly changing compounds in your dataset. In this particular case, the software was used for drift time alignment, peak picking, and normalization using total ion intensity. We obtained three data matrices, one for each of the investigated data set. Multiple features with same drift time and different *m/z* may belong to the same compound due to the fragmentation, adduct formation, or clustering. The three data matrices were then exported from Progenesis QI to the statistical package EZinfo (V3.0.1.0, Umetrics). This was used to build 2-class orthogonal projection to latent structure-discriminant (OPLS-DA) models and S-plots for each sample set under investigation. Protein Prospector V5.22.1 (UCSF Mass Spectrometry Facility) and Fragment Ion Calculator (ISB Data Access Server) were used to analyze the MS/MS fragmentation ions from peptides.

## Results

### ESI-IM-MS analysis revealed that Aβ40 predominantly oligomerizes through dimers and trimers

**Fig. 2:**
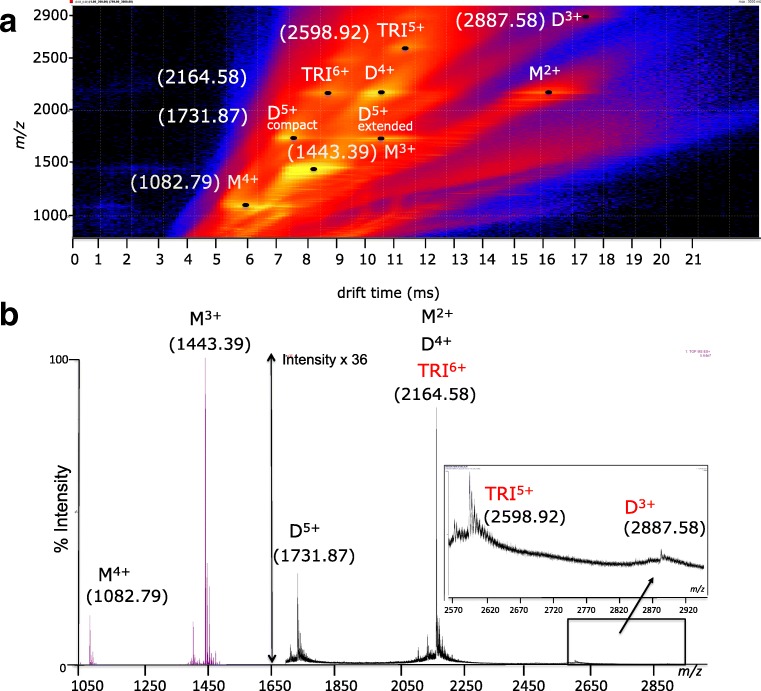
Aβ monomers in rapid equilibrium with low-order Aβ oligomers. At the top **a** 2D DriftScope IM-MS plot shows IM drift time versus *m/z* versus intensity. The signal amplitude is color-coded, increasing from purple (low intensity) to bright yellow (high intensity). The plot shows the Aβ40 species detected 5 min after diluting monomeric Aβ40 to the final peptide concentration of 20 μM. The peak apexes have been accentuated by dots and annotated as monomers, dimers, and trimers of Aβ40 marked with M, D, and TRI with their charge (protonation) states. The peaks assigned to the M^4+^, M^3+^, D^5+^, M^2+^, D^4+^, and TRI^+6^ are labeled with the corresponding experimental monoisotopic *m/z* (in parenthesis). The peaks assigned as TRI^5+^ and as D^3+^ are labeled with the experimental average *m/z* (in parenthesis). At the bottom **b** ESI-MS spectrum of 20 μM Aβ40. In red, the species detected with a S/*N*< 3. Satellite peaks observed correspond to alkali metal adduct commonly observed in ESI-MS

In agreement with previously reported data [25, 38, 39], the ESI-IM-MS analysis (Fig. 2) reveals that in the range 5–20 μM, the initially monomeric Aβ40 (M) oligomerizes predominantly through dimers (D) and trimers (TRI), the latter detectable with a signal-to-noise ratio lower than three (S/*N*<3). Peak assignments were performed as described in the Electronic Supplementary Material (ESM). As a result, the signal with a monoisotopic (mon) *m/z* at 1082.79 was assigned to [M+4H]^4+^; the signal at 1443.39 (mon) to [M+3H]^3+^; the three signals with the same (mon) *m/z* at 2164.58 were assigned to the [M+2H]^2+^, [D+4H]^4+^, and [TRI+6H]^6+^, respectively; the signal at average m/z 2598.92 was identified as [TRI+5H]^5+^; the signal at average *m/z* 2887.58 as [D+3H]^3+^. The two signals with the same (mon) *m/z* at 1731.87 but different mobilities (dt) were attributed to the compact and extended forms of the [D+5H]^5+^.

**Fig. 3:**
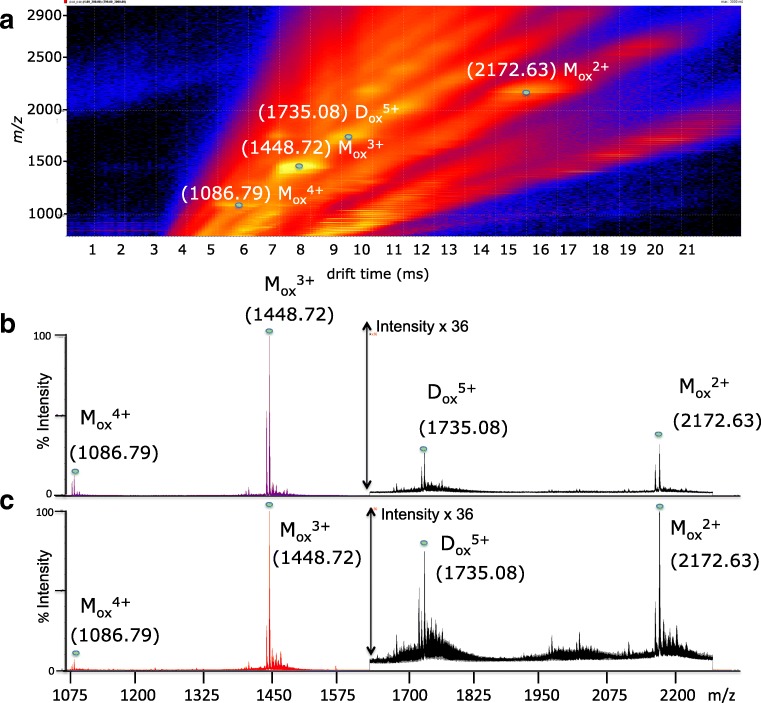
IM-MS of “Aβ40 plus Porph” sample sets (Aβ40:Zn-Porph, 1:1) revealed the detection of new four signals. **a** 2D DriftScope IM-MS plots of the “Aβ40 plus Porph” sample at 20 μM. Peak apexes have been accentuated by dots denoting the new detected species. Numbers in parenthesis above peaks denote their experimental monoisotopic *m/z* with the positive charge state of the ions given as a superscript. The species with *m/z* at 1448.72, at 2173.63, and at 1735.08 are also labeled with identity tag assigned following the considerations described in the following paragraph. The species at 1448.72 and at 2173.63 were assigned to the protonated Aβ40 oxidized monomer, triply (Mox^3+^), and doubly (Mox^2+^) charged, respectively, while the species with *m/z* at 1735.08 was identified as the quintuply protonated dimer of Aβ40 consisting of one unit of oxidized Aβ40 and another of unmodified Aβ40 (Dox^5+^). **b** ESI-MS spectrum of an “Aβ40 plus Porph” sample at 5 μM. **c** ESI-MS spectrum of an “Aβ40 plus Porph” sample at 20 μM. Satellite peaks observed correspond to alkali metal adducts commonly observed in ESI-MS

Upon the addition of the inhibitor (Aβ40: Zn-Porph, 1:1), the IM-MS of the “Aβ40 plus Porph” sample sets revealed the detection of four new signals (Fig. 3) with monoisotopic (mon) *m/z* at 1086.79, 1448.72, 1735.08, and at 2172.63, respectively. All of these peaks were associated to the corresponding charge state (see ESM Fig. S3). Furthermore, the signals of the Aβ40 species previously detected in the “Aβ40” sample sets (Fig. 2) were below the detection limit or reduced. Moreover, no other ATD peaks with S/*N*>3 and the same isotopic envelope of the two major [D+5H]^5+^ conformations were detected at drift times close to their drift times of the two major conformations.

### Identification of the new detected species

MS/MS experiments were performed to identify the four new species shown in Fig. 3. We first investigated the fragmentation pattern (see ESM Fig. S4) of the predominant monomeric M^3+^ species of Aβ40 with (mon) *m/z* at 1443.39 detected in “Aβ40” sample sets (Fig. 2). We mostly observed doubly and triply charged *b-type* ions covering the residues 11–39. MS/MS fragmentation patterns were then predicted with the use of MS/MS database search programs (or and compared with those detected in the MS/MS spectrum of the M^3+^ (see ESM Table S1). The MS/MS pattern of the M^3+^ of Aβ40 detected in the “Aβ40” sample sets was compared with the MS/MS patterns of the new species with *m/z* at 1086.79 (+ 4), 1448.72 (+ 3), 1735.08 (+ 5), and at 2172.63 (+ 2) observed in the “Aβ40 plus Porph” sample sets. Within the *m/z* range 200–1250, the MS/MS spectra of these species were the same as the MS/MS spectrum of the M^3+^ at *m/z* 1443.39. In contrast, the *m/z* range 1250–1420 indicated that the new detected species resulted from a partial or a total methionine oxidation at position 35 (Met-35(O)). Starting from the residue at the position 35, the fragments b_35_^3+^, b_36_^3+^, b_37_^3+^, b_38_^3+^, b_39_^3+^ previously detected for M^3+^ were shifted by 16.00 mass units (see ESM Fig. S5). This proves that the signals with (mon) *m/z* at 1448.72 and at 2173.63 correspond to the monomer triply—[Aβ40 Met-35(O)^+^3H]^3+^—and doubly [Aβ40 Met-35(O)+2H]^2+^ charged ions, respectively. The detection of b-fragments of both oxidized and not oxidized Aβ40 at the position 35–39 strongly suggests that the species at *m/z* 1735.08 is the dimer (+ 5)—[(Aβ40)(Aβ40 Met-35(O))+5H]^5+^—consisting of one unit of Aβ40 Met-35(O) and another of unmodified Aβ40 (see ESM Tables S2 and S3). The MS/MS spectrum of the species with *m/z* at 1086.79 (+ 4) was not covered by the *m/z* informative range 1250–1420; however, the comparison of the experimental versus theoretical value indicates it corresponds to the monomer quadruply charged, [Aβ40 Met-35(O)+4H]^4+^(see ESM Table S2).

### Multivariate analysis

**Fig. 4:**
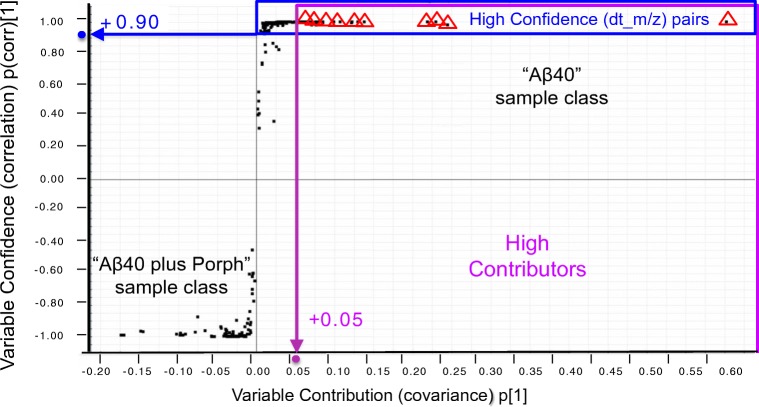
Representative S-plot of OPLS-DA model separating peptide profiles of “Aβ40” class and of the “Aβ40 plus Porph” class. The upper right quadrant of the S-plot shows those features which are elevated in “Aβ40” control group, while the lower left quadrant shows the pairs elevated in “Aβ40 plus Porph” class. The farther along the x-axis, the greater the contribution to the variance between the groups, while the farther the y-axis, the higher the reliability of the analytical result. The points are the features (drift time_ *m/z* pairs). *dt_m/z marker pairs* are selected based on their intra-class intensity variability, measured by the correlation parameter, p(corr)[1], as well as based on their contribution to the inter-class separation measured by the covariance parameter, p[1] shown in S-plot. p(corr)[1] > + 0.9 and p[1] > + 0.05 was employed as the threshold confidence interval

The effect of Zn-Porph on the early assembly of Aβ40 was studied using MVA [42, 43]. Each IM-MS run was imported into the Progenesis QI software as an ion intensity map including m/z and drift times. The software was used for IM-MS data pre-processing, specifically for drift time alignment, peak picking (see ESM Fig. S6), and normalization. Data matrices were then exported from Progenesis QI to the statistical package EZinfo. The MVA revealed significant features (dt_*m/z* pairs) in the “Aβ40” class whose signal intensity was reduced in the “Aβ40 plus Porph. 2-class OPLS-DA models [46], built on the entire ATDs, were constructed for each dataset to compare the peptide profiles detected for the “Aβ40” sample class to those detected for the “Aβ40 plus Porph” class. Pareto scaling was used for the model construction. All OPLS-DA models had high value (0.99) of the *goodness-of-fit* parameter R2Y (cum). R2Y (cum) indicates how well the model fits data, as total sum of variation in Y explained by the model. S-plots of 2-class OPLS-DA models were constructed for each dataset. High discriminatory dt_*m/z* pairs of the “Aβ40” class, and hence, with likelihood of being potential-robust quality markers (from now *dt_m/z marker pairs*), were automatically selected on the basis of their intra-class intensity variability, measured by the correlation parameter, p(corr)[1], as well as of their contribution to the inter-class separation, measured by the covariance parameter, p[1]. p(corr)[1] > + 0.9 and p[1] > + 0.05 was the confidence interval adopted as threshold according to the selection procedure illustrated in Fig. 4.

**Fig. 5:**
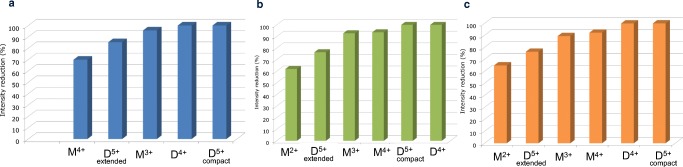
The blue (**a**), the green (**b**), and the orange (**c**) histograms show the inhibitory effect of Zn-Porph (added to monomeric Aβ40 in DMSO in a 1:1 M ratio) on the formation of soluble monomeric and dimeric species of Aβ40 selected as *dt_m/z marker pairs* for the sample sets at 5 μM (**a**), 20 μM (**b**), and 20 μM incubated before injection (**c**). The height of each bar represents the reduction (in %) of the average ion intensity of each *dt_m/z marker pair* measured in the “Aβ40 plus Porph” sample class with respect to the average intensity measured in the “Aβ40” class. The species M^4+^(6.28_1082.79), M^3+^(7.83_1443.39), D^5+^compact (7.28_1731.87) conformation, D^5+^extended (10.80_1731.87) conformation, and D^4+^(10.36_2164.58) were all selected in the three sample sets as *dt_m/z marker pairs* to evaluate the inhibitory effect of Zn-Porph on their formation*.* In the sample set at 5 μM (**a**), the feature M^2+^(16.10_2164.58) was instead excluded due to its high intra-class intensity variability (p(corr)[1] < + 0.9)

The selected *dt_m/z marker pairs* of each sample set were finally ranked using the variable importance in projection (VIP) [48] plots (see ESM Fig. S7) to speed up the interpretation of the results. We observed that in all the sample sets under investigation, due to the low signal-to-noise (S/N) and the poor repeatability of the ion intensities (p(corr)[1] < + 0.9), the feature**s** corresponding to the TRI^6+^, TRI^5+^, and to the D^3+^ of Aβ40 were automatically excluded (VIP < 1). The feature corresponding to M^2+^ with a dt_*m/z* pair of 16.10_2164.58—labeled as M^2+^(16.10_2164.58) pair—was excluded only in the sample set at 5 μM, while it was selected as robust quality marker in the two sample sets at 20 μM. The features M^3+^(7.83_1443.39), M^4+^(6.28_1082.79), and D^4+^(10.36_2164.58), (10.80_1731.87), and (7.28_1731.87) corresponding to the extended and compact conformations of the D^5+^ were selected as *dt_m/z marker pairs* to evaluate the inhibitory effect of Zn-Porph on their formation in the three sample sets investigated. By clicking on the VIP plot’s bars (see ESM Fig. S8) corresponding to *dt_m/z marker pairs* with a VIP value higher than one, ion intensity trend plots (XVar trend plots) were quickly generated to visualize the measured ion intensity value of each pair across all samples of the “Aβ40” and “Aβ40 plus Porph” sample classes (see ESM Fig. S9). The average ion intensity of each *dt_m/z marker pair* in the two classes (see ESM Tables S4, S5, S6) was finally used to estimate the in vitro inhibition by the Zn-Porph on their formation. This was expressed as the reduction (in %) of the average ion intensity of each *dt_m/z marker pair* measured in the “Aβ40 plus Porph” class with respect to the average value measured in the “Aβ40” class (Fig. 5). Our findings revealed that in all data sets investigated, Zn-Porph alters the distribution of both monomeric and dimeric conformers of Aβ40 over the timescale of our experiments. In particular, at 20 μM, the abundance of the conformer M^2+^(16.10_2164.58) is reduced by 62–65% compared with that observed for Aβ40 alone. The ATD peaks of the monomeric species corresponding to the features M^4+^(6.28_1082.79) and M^3+^(7.83_1443.39) significantly diminished compared with Aβ40 alone with a reduction of the monomeric conformer M^4+^ between 92 and 94% in the sample sets at 20 μM and of 70% in the sample set at 5 μM. The ESI-IM-MS data provided us with the evidence that Zn-Porph inhibits or eliminates the formation of Aβ40 homodimers. Upon the addition of the Zn-Porph, the ATD features of the compact conformation D^5+^(7.28_1731.87) and of D^4+^(10.36_2164.58) were indeed essentially eliminated, while the ATD peak of the extended conformation D^5+^(10.80_1731.87) was significantly diminished (by 85% in the sample set at 5 μM and by 76% in the two sets at 20 μM).

## Discussion

A key aspect of the present study is the introduction of IM-MS-MVA as a novel approach to perform data analytics in drug discovery/amyloid research targeting the in vitro aggregation of synthetic Aβ peptide at low concentrations. The in vitro Aβ self-assembly studies are challenging due to the non-reproducible behavior of synthetic Aβ. To start with, Aβ normally circulates in plasma and cerebrospinal fluid in nanomolar to picomolar concentrations [49–51], and the in vitro dynamics are highly dependent on its concentration [52], manufacturing route (synthetic or recombinant), methods of synthesis, and purification [53]. Additionally, the discordance between previous findings suggests that small variations in the environmental conditions and/or subtle chemical changes during the dissolution step [54, 55] may lead to differences in the size and conformational distribution of LMWs, especially as it has been reported as several different polymorphs exist [56, 57]. For the first time, we systematically analyzed the IM-MS profiles of LMWs of Aβ40 peptide (Aβ40 LMWs) in vitro models prepared from eight independent dissolutions of synthetic peptide, these are grouped as “Aβ40” sample class. Three sample sets were investigated, specifically, at 5 μM, 20 μM, and at 20 μM incubated at 37 °C for 2 h prior to injection. Using IM-MS-MVA, we investigated the inhibitory effect of the anti-fibrillogenic Zn-Porph on Aβ40 LMWs previously detected in the “Aβ40” sample class. Specifically, for each data set, MVA was used to remove the ATD peaks with high intra-class ion intensity variability in the “Aβ40” sample class as well as figured out the peaks that were significantly affected by the inhibitor in the “Aβ40 plus Porph” class. Our study revealed that in the “Aβ40” sample class, the initially monomeric (M) Aβ40 peptide oligomerizes predominantly through dimer (D) and trimer (TRI), the latter detectable with a S/*N*< 3. It is important to note that our results are consistent only with those studies that used the ^13^C isotope distribution to assign peaks separated in the mobility dimension [25, 35]. Of note, IM-MS data of the “Aβ40” sample class shows multiple monomer charge states, predominantly triply and quadruply charged. As previously reported by Young and co-workers [38] for the monomeric human islet amyloid polypeptide (hIAPP), and by Dadlez and co-workers [35] for the monomeric Aβ40, the distinct monomeric charge states may be indicative of a change in shape due to unfolding during the oligomerization process. Unfolded (extended) monomeric conformations expose more ionizable sites and thus give rise to higher charge states during ESI than the folded conformations of the same peptide. In IM-MS ion intensity, quantification involves the integration of the area under the ATD peak, in the drift time spectra and mass spectra, and intensity is used as a direct measure of peptide abundance. Direct comparison of the corresponding peptide ATD peak area across different samples allows for the relative quantification of peptides. We initially observed that comparison of ATD peaks of the Aβ40 species within the “Aβ40” sample class was complicated by ATD drifts. These can be due to a host of different factors, including sample stability, temperature and pressure fluctuations, deposit build-up, and heterogeneity and dynamic nature of Aβ40 peptide and of its LMWs. The presence of interferences, e.g., other analytes with a similar ion mobility, and changing of peak positions dependent on environmental conditions, e.g., in the field operations, could strongly hamper proper analyte identification and quantification. For the purpose of this study, we focused on the major species detected in “Aβ40” sample class that give distinguishable peaks in the extracted ATDs (see ESM Fig. S2). In this respect, as previously reported [58], the structural heterogeneity and dynamic nature of the D^+5^ species is reflected in their ATD peak shape. ATD peaks that deviate slightly from Gaussian are consistent with multiple states, indicating the existence of multiple conformers rapidly interconverting on the ESI-IM-MS timescale [58]. We adopted an automatic alignment procedure, which compensates for small variation between runs in the IM drift times to combine and compare IM-MS profiles of Aβ40 early species from different dissolutions without ATD peak distortions (see text in ESM). Using the automatic alignment tool, frames detected in all runs are automatically aligned with the base frames detected in a sample of the “Aβ40” sample class, selected as the reference run. Alignment is an essential 1-D IM-MS data pre-processing step before MVA to achieve IM spectra that are reproducible between different samples and conditions [43]. It corrects for small variations in the temperature and pressure of the drift tube, resulting in changes in analyte drift time. This results in an increased precision of the ion-abundance measurement of a peptide feature (dt_*m/z* pair) across multiple runs. Although the drift time alignment scores are dependent on the degree of overlap between features, and misalignment of conflicting features may still yield positive alignment scores, we used these scores as a qualitative measure of the IM-MS alignment, along with visual interpretation to determine alignment success. All 16 samples from the two sample classes were determined to have good alignment scores (> 80%). From these analyses, we conclude that drift time alignment scores should be at least > 80%, and ideally > 90%, to minimize variation and improve precision in ion-abundance measurements. This process is important because it facilitates consistent peak picking across multiple runs, enables appropriate normalization of data, reduces complications in assigning peptide identity, and allows the direct comparison of Aβ40 LMW features across the “Aβ40” and “Aβ40 plus Porph” sample classes (see ESM Fig. S6). This strategy is not only relevant to Aβ40 assembly but may be useful to the studies focusing on the inhibition on Aβ42 assembly and on other aggregation diseases such as Parkinson (PD) or amyotrophic lateral sclerosis (ALS). Our findings revealed that in all data sets investigated, Zn-Porph alters the distribution of both monomeric and dimeric conformers of Aβ40 over the timescale of our experiments, inhibiting their formation at the early stage of the aggregation pathway of Aβ40. However, no complexes of Aβ40 and Zn-Porph were observed. In this regard, it is important to note that the ESI process does not maintain hydrophobic interactions in the gas-phase for very large molecular weight complexes [59], especially, when the ligand has a molecular weight higher than 800 Da, as in the case of Zn-Porph. The correspondence of MS/MS patterns predicted using de novo peptide sequencing algorithms to those ones of the species at *m/z* 1448.72 (+3), 1735.08 (+5), and at 2172.63 (+2) detected in the “Aβ40 plus Porph” sample sets detected in the “Aβ40 plus Porph” sample classes (see ESM Table S3) suggested a porphyrin-catalyzed oxidation in position 35 (Met-35(O)). This led to the detection of Aβ40 Met-35(O) monomer and mono-oxidized dimer; the latter consisting of one unit of Aβ40 Met-35(O) and another of unmodified Aβ40—[(Aβ40)(Aβ40 Met-35(O)]. The detection of one major ATD peak for the mono-oxidized dimer (Dox^5+^) with *m/z* (mon) at 1735.08 and with discernable isotopic distribution pattern (Fig. 3 and ESM Fig. S3) was an indication that the oxidation could possibly occur at Met-35 on one of the two D^+5^ conformers. A previous study [60] conducted on synthetic Aβ40 by the sensitive electrospray ionization Fourier transform ion cyclotron resonance mass spectrometry (ESI-FTICR-MS) has shown that spectra acquired between 20 and 30 min immediately after dissolving the peptide contained less than 1% of Aβ40 Met-35(O). We, thus, exclude that the species detected in the “Aβ40 plus Porph” sample classes could be a result of a spontaneous Aβ40 oxidation over the timescale of our experiments. We also exclude that these could be oxidation artifacts of the ESI process [61] having used gentle ESI conditions (the capillary voltage and cone voltage were 2.8 kV and 38 V, respectively). Our results therefore strongly suggest a porphyrin-catalyzed oxidation at the position 35 following the dilution of monomeric Aβ40 peptide with acetate buffer containing an equimolar amount of Zn-Porph. Such event is not unexpected since, as previously reported, porphyrin and its analogues have exhibited catalytic activity for highly selective mono-oxygenation reactions which proceed through singlet oxygen (^1^O_2_) generation [62–65]. The generated singlet oxygen readily reacted with the unoxidized Aβ40 leading to the detection of the new detected species caused by the oxidation of Met-35. It has been suggested that oxidation of Met-35(O) in Aβ peptides significantly inhibits fiber formation. In vitro oxidation of Aβ, by the physiological oxidant hydrogen peroxide (H_2_O_2_), was monitored using Thioflavin-T (ThT), transmission electron microscopy (TEM) [66], circular dichroism (CD) [67], and solution NMR [68]. All of these studies suggested a disrupting effect of Met-35(O) on β-sheet formation. In particular, solution NMR findings indicate that Met-35(O) prevents aggregation by reducing both hydrophobic and electrostatic association and that the unoxidized and oxidized Aβ peptides may associate differently, through specific, sharp changes in structure during the initial stages of aggregation [69]. This raises the question of whether the porphyrin-catalyzed oxidation positively affects the complex early assembly of Aβ40 oligomers that ultimately lead to the fibers and plaques in the brain. ESI-FTICR-MS has shown that Aβ methionine in vitro oxidation induced by H_2_O_2_, which is a relatively mild oxidant present physiologically, inhibits trimer but not dimer formation in the early stage of Aβ40 aggregation [47]. In this study, H_2_O_2_ was added to a final concentration of 2.7% to synthetic Aβ40 freshly dissolved in deionized water/acetic acid 99:1 (v/v), pH 3 to a final peptide concentration of 4 μM. Our IM-MS-MVA combined study clearly provide us with the evidences that upon addition of the Zn-Porph, the ATD features of the compact conformation D^5+^and of D^4+^ were essentially eliminated, while the ATD peak of the extended conformation D^+5^ was significantly diminished. The latter by 85% in the sample set at 5 μM and by 76% in the two sets at 20 μM. Our explanation is related to the possibility that Zn-Porph more likely forms a complex with the compact conformer being involved in an on-pathway fibrillation process, whereas the extended one could be involved also to the off-pathway fibrillation leading to amorphous aggregates. Discordance between our findings and those from FTICR-MS can be reconciled by taking into account the different oxidant involved in the process, H_2_O_2_ and aerobic oxygen, respectively. In this framework, a research group at the University of Tokyo recently found that catalytic oxygenation of Aβ peptides might be an effective approach to treat AD [69]. In this study, oxygenation of Aβ by flavin catalyst attached to an Aβ-binding peptide induced two favorable features for the treatment of AD. First, the pathological properties of native Aβ, aggregation potency and neurotoxicity, were markedly attenuated by oxygenation. Second, the oxygenated Aβ inhibited the aggregation and cytotoxicity of native Aβ. Thus, flavin-catalyzed photo-oxidation of Aβ not only decreases the concentration of aggregative and pathogenic natural Aβ, but also increases the concentration of an aggregation inhibitor (oxygenated Aβ).

## Conclusions

An attractive therapeutic approach for AD treatment is to remodel the initial stages of Aβ assembly in a way that attenuates the neurotoxicity of the transient LMWs. Furthermore, the integration of therapeutic moieties and diagnostic ones in the same chemical scaffold a step forward towards personalized medicine for AD. ESI-IM-MS combined to MVA revealed that in all sample sets, the Zn-Porph alters the distributions of both monomeric and dimeric conformers of Aβ40 inhibiting their formation at the early stage of the Aβ40 aggregation pathway. The correspondence of the MS/MS patterns predicted using MS/MS database search programs to those ones of the species detected in the samples of Aβ40 containing Zn-Porph suggested a porphyrin-catalyzed oxidation at Met-35(O) of Aβ40. This led to the formation of Aβ40 Met-35(O) monomer and mono-oxidized dimer consisting of one unit of Aβ40 Met-35(O) and another of unmodified Aβ40. Furthermore, our previously conducted MALDI-ToF-MS experiments indicated that Zn-Porph is able to aggregate, via formation of supramolecular adduct, with the monomeric Aβ42. Thus, binding to and stabilizing Aβ40 monomer, with concomitant catalyzed oxidation, could be the mechanism of the A*β* self-assembly inhibition by this candidate theranostic agent. The Zn-Porph investigated here could indeed potentially serve as in-vivo fluorescent ligand for visualization and identification of soluble LMWs of Aβ in biological fluids, progression prediction, and differential diagnosis, to finally tailor personalized and precision dosages [70–74]. This remains the big challenge in AD drug discovery. Going forward, intrinsic porphyrin bio-compatibility and multimodality will keep new applications of this class of molecules at the forefront of theranostic research [75, 76]. We here also introduced a novel data analytics approach in drug discovery/amyloid research. This systematic approach could be particularly suitable in amyloid research aiming at evaluating the inhibitory effect of a candidate AD drug on the early-assembly of Aβ in vitro models at very low concentration levels of Aβ.

## Electronic supplementary material

ESM 1 (PDF 4434 kb)
