# Supplementary material for: Ion mobility spectrometry combined with multivariate statistical analysis: revealing the effects of a drug candidate for Alzheimer’s disease on Aβ1-40 peptide early assembly
Source: Anal Bioanal Chem. 2019 Aug 12;411(24):6353–63. doi: 10.1007/s00216-019-02030-7 (PMC6718366; doi:10.1007/s00216-019-02030-7)
Supplement: Supplementary file 1 — (PDF 4434 kb) [file 216_2019_2030_MOESM1_ESM.pdf]

Analytical and Bioanalytical Chemistry

Electronic Supplementary Material

Ion mobility spectrometry combined with multivariate statistical analysis: revealing the effects of a drug candidate for Alzheimer's disease on Aβ1-40 peptide early assembly

Serena Lazzaro, Nina Ogrinc, Lieke Lamont, Graziella Vecchio, Giuseppe Pappalardo, Ron M.A. Heeren

Assignment of the ESI-IM-MS spectra to specific early-stage species of Aβ40

Aβ native monomer in a freshly prepared solution exists in rapid equilibrium with lowly populated and short-lived low molecular weight oligomers (LMWs) further evolving into higher order aggregates. Using gentle ESI conditions, small aggregates of Aβ in water can be transferred to the gas phase, making it possible to “freeze” by IM-MS the size and conformational distribution of the transient and not-covalently-stabilized LMWs. Drift time (dt) measurement allows the IMS of the species with the same mass -such as compact and extended conformations of D^{5+}- or with the same *m/z* value -such as for the protonated monomer (M^{2+}), dimer (D^{4+}) and trimer (TRI^{6+}). These could not be identified in the classic MS spectrum due to their overlapping isotopic envelope (Fig. 2,b). By contrast, these can be seen as separate species due to their different mobility (dt), as they drift through a neutral gas under the influence of a weak electric field. LMWs with compact conformations undergo fewer collisions with neutral gas than those with extended conformations, and thus will have higher mobilities (<dt). Similarly, LMWs with higher charge states will also have higher mobilities. As result, the size and conformational distribution of species can been fixed on a two-dimensional map (Fig. 2,a), with signals separated both in the domain of *m/z* and in the dt, as previously described[35]. In our work, peak assignments were performed using their ^{13}C isotope distributions of the protonated species separated in the IM dimension with the MS operating in resolution mode. Based on their resolved isotopic distribution (Fig. S1), the signal with a monoisotopic (mon) *m/z* at 1082.79, consistent with a charge +4, was assigned to M^{4+}; the signal at 1443.39 (mon), consistent with charge +3, to M^{3+}; the three signals with the same (mon) *m/z* at 2164.58, consistent with charge +2, +4 and +6, were assigned to the M^{2+}, D^{4+} and TRI^{+6}, respectively; the signal at average *m/z* 2598.92, consistent with a charge state +5, was identified as TRI^{5+}; the signal at average *m/z* 2887.58 as D^{3+}. The conformation of the ions was also taken into account for the assignment of the two species with same (mon) *m/z* at 1731.87 and ^{13}C isotope distribution. These species, having different mobilities (dt), were attributed to the compact and

extended forms of the D^{5+}. The D^{5+} with compact conformation undergoes fewer collisions with neutral gas as it drifts under the influence of the electric field than that one with extended conformation. As result, compact D^{5+} will have higher mobility (<dt) as shown on Fig. 2,a and Fig. S2,d.

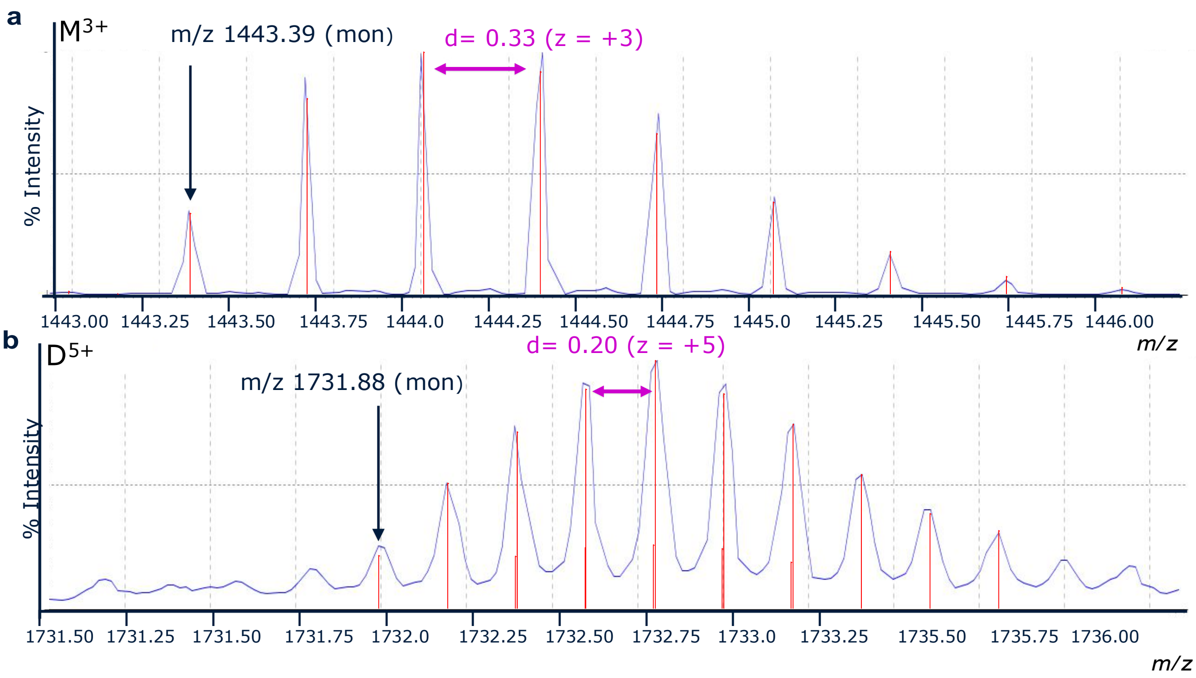

Fig. S1 Representative ^{13}C isotope distributions of the Aβ40 protonated species separated in the IM dimension. These were used to assign the charge state to each species detected in "Aβ40" sample-sets. Isotopic envelopes were extracted from the 2D Driftscope IM-MS plot (shown on Fig. 2,a). Each signal is labelled with the associated experimental monoisotopic (mon) *m/z*, highlighted by arrows, and the measured Da spacing (d, in purple) of the isotopic envelope, indicating the corresponding charge state (z). The signal with (mon) *m/z* at 1443.39 (panel a) was therefore assigned to the quadruply-protonated monomer of Aβ40 (M^{4+}), while the two signals with (mon) *m/z* at 1731.87, having the same envelop (panel b) but different drift times (at 7.28 and 10.80 ms, as shown on Fig. 2,a and Fig.S2,d) were assigned to the quintuply-protonated Aβ40 dimer (D^{5+}), as compact and extended conformations, respectively

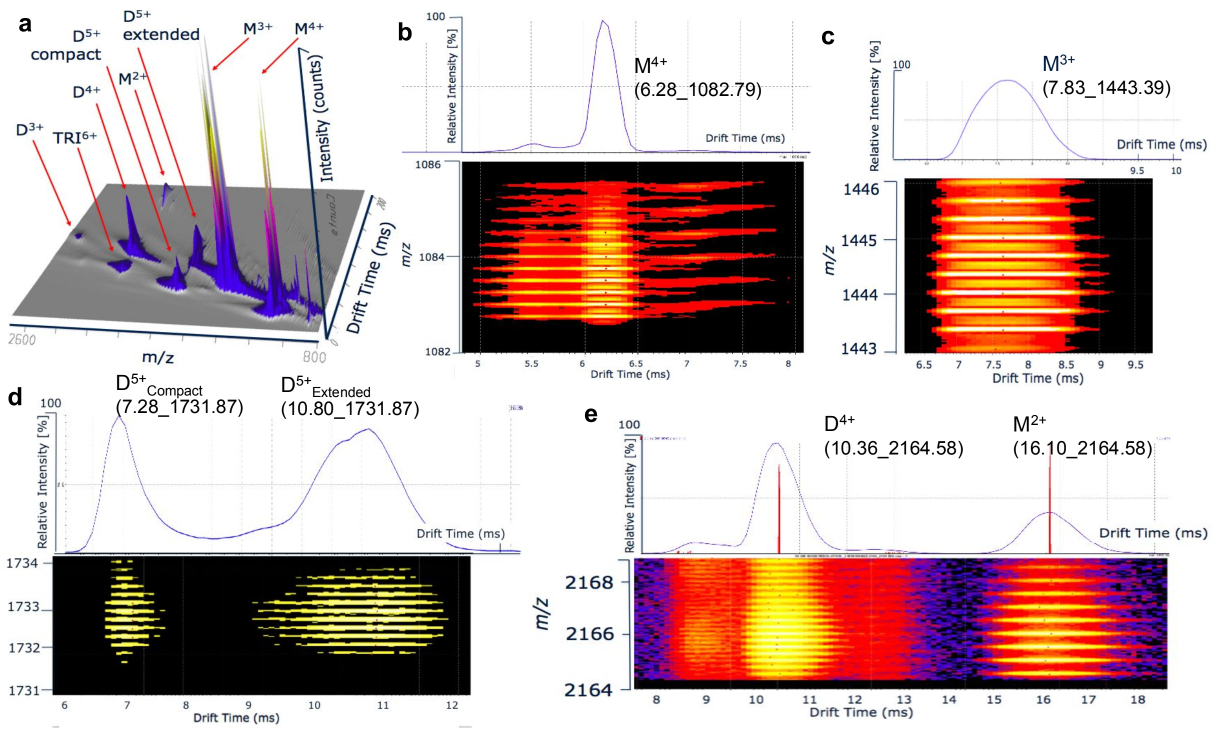

Fig. S2 Enlarged regions selected on the 2D Driftscope IM-MS plot (shown on Fig. 2,a) covering the signal of the quadruply (M^{4+}, inset b) and triply-protonated (M^{3+}, inset c) monomer of Aβ40, the quituply-protonated dimer, as compact and extended conformations (D^{5+}, inset d) –these overlapping in the ESI-MS spectrum (Fig. 2,b)-, and the quadruply-protonated dimer and doubly-protonated monomer (D^{4+} and M^{2+}, inset e) overlapping as well in the ESI-MS spectrum. The colored spots indicate MS peaks with amplitude increasing from purple to yellow. All signals can be well resolved in the 3D Driftscope IM-MS plot (inset a) into mobility peaks with different dt and arrival time distribution areas (ATDs). For each of the detected ATD peak, their associated dt (taken from the vertical line at the apexes of the ATD area, shown as an example on inset e) and their monoisotopic *m/z* (highlighted by arrows on Fig. S3) is shown as dt_*m/z* pair label at the top of each peak in the extracted ion mobilogram

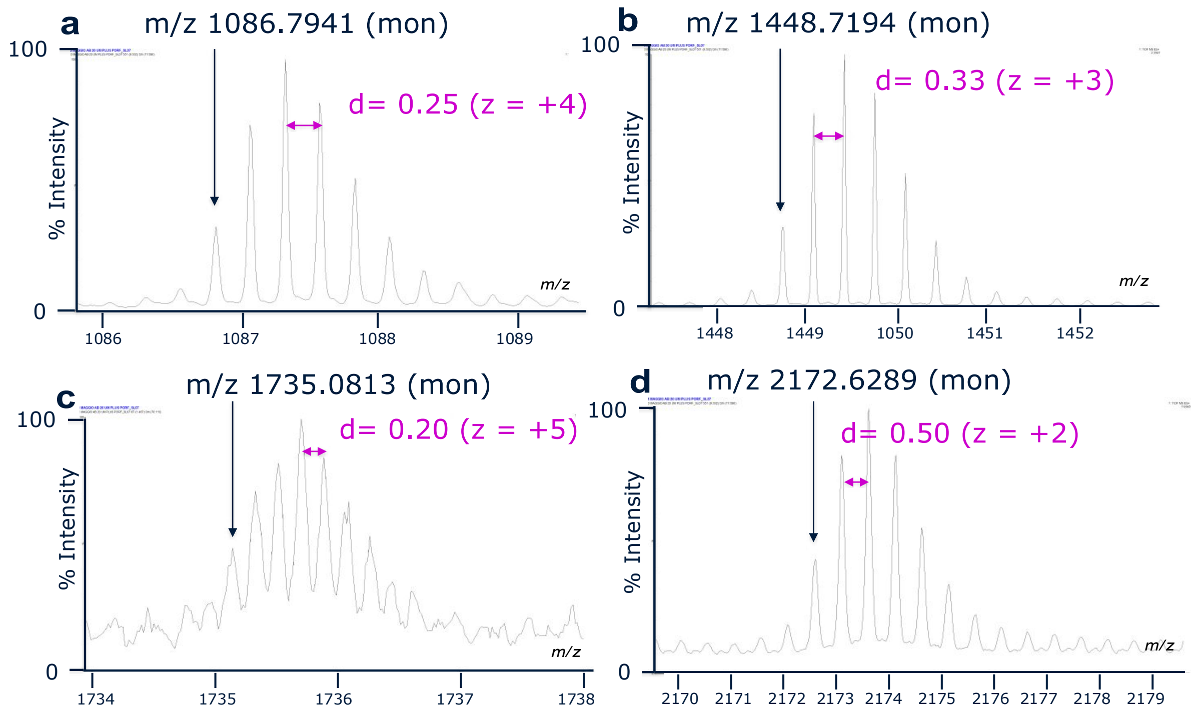

Fig. S3 Isotopic envelopes of the protonated species detected in "Aβ40 plus Porph" sample-sets (Aβ40:Zn-Porph, 1:1). Each signal is labelled with the associated experimental monoisotopic (mon) *m/z*, highlighted by arrows, and the Da spacing (d, in purple) of the isotopic envelope, indicating the corresponding charge state (z)

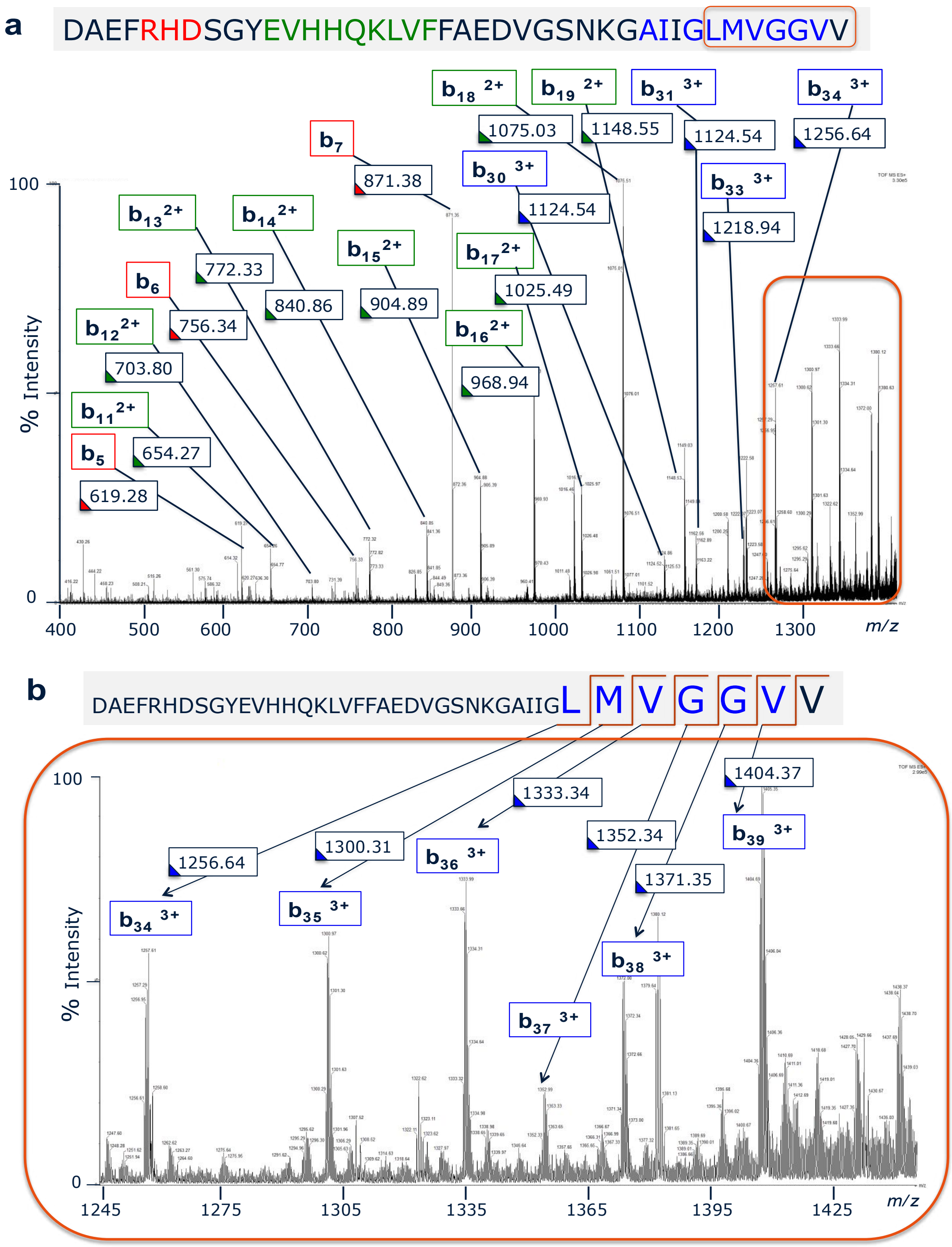

Fig. S4 At the top, MS/MS fragmentation spectrum (CE 70) of triply-protonated monomer of Aβ40 (M^{3+}) with (mon) *m/z* at 1443.39 detected in "Aβ40" sample-sets. Data were acquired within the *m/z* range 200-1440 applying in the TRAP cell a collision energy (CE) value of 70 eV with the quadrupole analyzer used to select ions through a narrow window (LM resolution window: 6.5). The inset in panel a is the full-length Aβ 1-40 amino acid sequence. The *b-type* ions are labelled with corresponding monoisotopic *m/z*. Singly charged product *b-type* ions are marked with red boxes, while the predominant doubly and triply charged *b-type* ions in

green and blue, respectively. b) At the bottom, the zoom into the MS/MS spectrum (shown in a) in the *m/z* range 1245-1440 covering the residues 34-39. Predominant triply charged *b-type* ions are labelled with blue boxes and corresponding monoisotopic *m/z*

Fragment	Expected (mon) *m/z*	Experimental (mon) *m/z*	Δ (ppm)
b_5^{1+}	619.2835	619.2847	1.9377
b_{11}^{2+}	654.2680	654.2657	3.5153
b_{12}^{2+}	703.8022	703.8019	0.4262
b_6^{1+}	756.3424	756.3454	3.9664
b_{13}^{2+}	772.3317	772.3304	1.6832
b_{14}^{2+}	840.8611	840.8630	2.2595
b_7^{1+}	871.3693	871.3778	9.7547
b_{15}^{2+}	904.8904	904.8896	0.8840
b_{16}^{2+}	968.9379	968.9412	3.4057
b_{17}^{2+}	1025.4799	1025.4896	9.4589
b_{18}^{2+}	1075.0141	1075.0258	10.8835
b_{30}^{3+}	1124.5325	1124.5444	10.5821
b_{19}^{2+}	1148.5483	1148.5532	4.2662
b_{31}^{3+}	1162.2271	1162.2291	1.72083
b_{32}^{3+}	1199.9218	1199.9303	7.0837
b_{33}^{3+}	1218.9290	1218.9454	13.4544
b_{20}^{2+}	1222.0825	1222.0861	2.9457
b_{34}^{3+}	1256.6237	1256.6354	9.3106
b_{35}^{3+}	1300.3038	1300.3066	2.1533
b_{22}^{2+}	1322.1224	1322.1283	4.4625
b_{36}^{3+}	1333.3266	1333.3383	8.7750
b_{37}^{3+}	1352.3338	1352.3353	1.1091
b_{38}^{3+}	1371.3410	1371.3538	9.3339
b_{23}^{2+}	1379.6359	1379.6400	2.9717
b_{39}^{3+}	1404.3638	1404.3707	4.9132
M^{3+}	1443.3901	1443.4110	14.4798

Table S1 Expected and experimental monoisotopic (mon) *m/z* value of *b-type* ions covering the residues 35-39 detected in the ESI MS/MS spectrum (Fig. S4). In column four the deviation is given (in parts per million) for the expected versus the experimental values

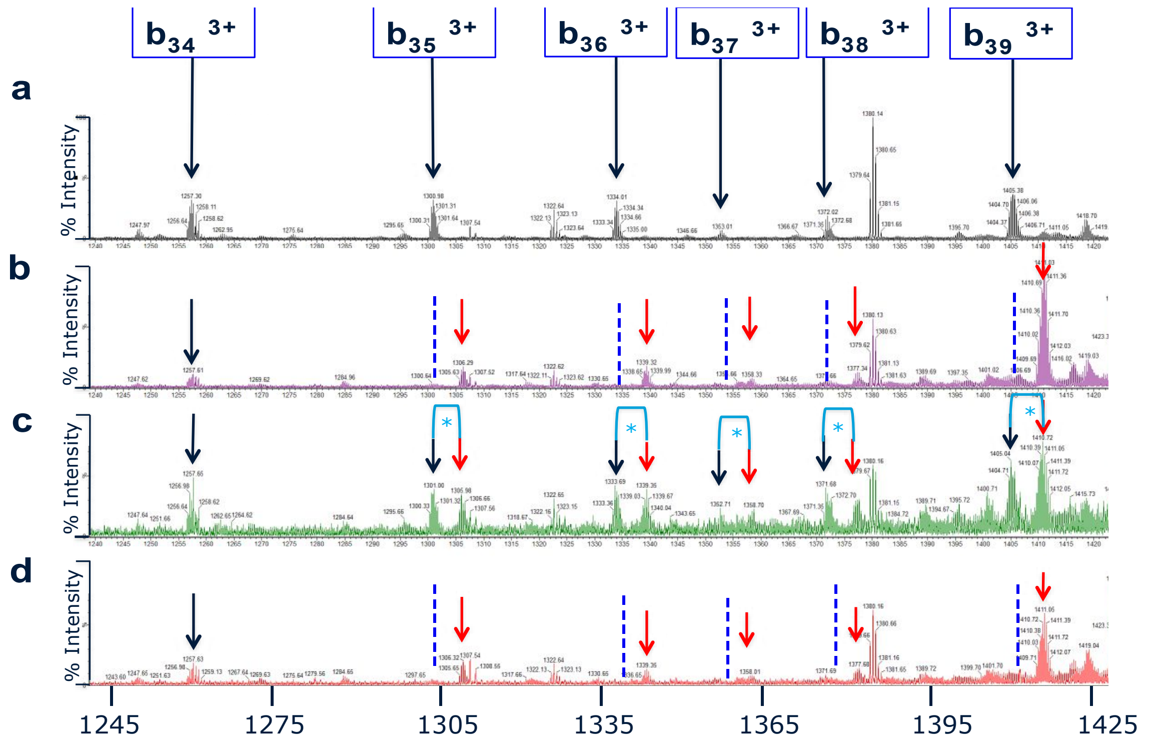

Fig. S5 Zoom of the 1245-1425 *m/z* range covering the C-terminal part of the Aβ40 sequence. Comparison of the MS/MS patterns of M^{3+} of Aβ40, with *m/z* (mon) at 1443.39, detected in the "Aβ40" sample-sets, to those of the species with *m/z* (mon) at 1448.72 (+3), 1735.08 (+5) and at 2172.63 (+2), detected in the "Aβ40 plus Porph" sample-sets. On the panel (a), highlighted with black arrows are the b_{34}^{+3}, b_{35}^{+3}, b_{36}^{+3}, b_{37}^{+3}, b_{38}^{+3} and b_{39}^{+3} detected for M^{3+} of Aβ40. Corresponding *m/z* values are shown on Fig. S 4,b. Starting from the residue at the position 35, the signals (marked with red arrows) were shifted by 16.00 mass units (*Δm) on the MS/MS spectra of the species with *m/z* (mon) at 1448.72 (+3) (panel b) and at 2172.63 (+2) (panel d). The missing signals, previously detected on the panel (a) are marked with blue dotted lines. This proves that the signals at *m/z* 1448.72 and at 2173.63 detected in the "Aβ40 plus Porph" sample-sets correspond to the monomer triply -[Aβ40 Met-35(O)+3H^+]$^{3+}$- and doubly [Aβ40 Met-35(O)+2H^+]$^{2+}$ charged ions, respectively. The co-detection of *b*-fragments of oxidized (marked with red arrows) and not oxidized (marked with black arrows) Aβ40 at the position 35-39 (panel c) is indicative that the species with *m/z* at 1735.08 is the dimer (+5) -[(Aβ40)(Aβ40 Met-35(O)) +5H^+]$^{5+}$- consisting of one unit of Aβ40 Met-35(O) and another of unmodified Aβ40

New detected species	Expected (mon) *m/z*	Experimental (mon) *m/z*	Δ (ppm)
[Aβ 40 Met-35(O)+4H^{+}]$^{4+}$	1086.7944	1086.7941	0.2760
[Aβ 40 Met-35(O)+3H^{+}]$^{3+}$	1448.7235	1448.7194	2.8300
[(Aβ40)(Aβ40 Met-35(O))+5H^{+}]$^{5+}$	1735.0656	1735.0813	9.04865
[Aβ 40 Met-35(O)+2H^{+}]$^{2+}$	2172.5815	2172.6289	21.8174

Table S2 Expected monoisotopic (mon) *m/z*, experimental monoisotopic *m/z* and the resulting mass error (ppm) calculated for the species with *m/z* (mon) at 1086.79(+4), 1448.72 (+3), 1735.08 (+5) and at 2172.63 (+2) detected in the three "Aβ40 plus Porph" sample-sets (Fig. 3)

Fragment	Expected (mon) *m/z*	MS/MS 1448.7 Experimental (mon) *m/z* [Aβ40 Met-35(O) +3H^{+}]$^{3+}$	Δ (ppm)	MS/MS 1735.1 Experimental (mon) *m/z* [(Aβ 40)(Aβ 40 Met-35(O)) +5H^{+}]$^{5+}$	Δ (ppm)	MS/MS 2173.6 Experimental (mon) *m/z* [Aβ 40 Met-35(O) +2H^{+}]$^{2+}$	Δ (ppm)
b_{34}^{3+}	1256.6237	1256.6210	2.1486	1256.6399	12.8917	1256.6364	10.1064
b_{35}^{3+}	1300.3039	absent	N/A	1300.3252	16.3808	absent	N/A
b_{35}^{3+} Met35(0)	1305.6372	1305.6307	4.9784	1305.6649	21.2157	1305.6466	7.1995
b_{36}^{3+}	1333.3267	absent	N/A	1333.3560	21.9751	absent	N/A
b_{36}^{3+} Met35(0)	1338.6600	1338.6492	8.0678	1338.6672	5.3785	1338.6812	15.8367
b_{37}^{3+}	1352.3338	absent	N/A	Low S/N	N/A	absent	N/A
b_{37}^{3+} Met35(0)	1357.6672	1357.6515	11.5640	1357.6854	13.4054	1357.6837	12.1532
b_{38}^{3+}	1371.3410	absent	N/A	1371.3541	9.5527	absent	N/A
b_{38}^{3+} Met35(0)	1376.6743	1376.6581	11.7675	1376.6758	1.0896	1376.6906	11.8401
b_{39}^{3+}	1404.3638	absent	N/A	1404.3864	16.0927	absent	N/A
b_{39}^{3+} Met35(0)	1409.6971	1409.6887	5.9587	1409.7224	17.9471	1409.7051	5.6750

Table S3 Mass error (ppm) calculated for monoisotopic (mon) ***b-type*** ions covering residues 35–39 in the MS/MS spectra of the species at *m/z* 1448.72 (+3), 1735.08 (+5) and at 2172.63 (+2) detected in the "Aβ40 plus Porph" sample-sets

Automatic alignment procedure

All IM-MS run belonging to the “Aβ40” sample-class and to the “Aβ40 plus Porph” class were imported to Progenesis QI software as ion-intensity maps including m/z and drift times. One acquisition of the “Aβ40” sample-class was manually selected as a reference run and by using the automatic alignment tool frames detected in all runs were automatically aligned. To ensure consistent peak picking and matching across all data files, an aggregate 2D IM-MS single map is created from the aligned runs. This map contains all peak information from all sample files. This map is then applied to each samples, and the alignment scores is shown for each of them. The map is then used for the peak picking in the two sample-classes, so that the same ions detected in the reference sample are detected in all runs. The peak-picking algorithm can discern overlapping peptide ions and retains information about the ATDs peak shape in each run. The automatic alignment procedure was assisted by a “review alignment” step enabling the visualization of a multi-panel window including a montage of 3D ion-intensity map (including m/z and drift times), 2D ion-mobilogram (intensity *vs* drift time) and the MS of each selected area on the map before and after the alignment. This panel helps to validate the ions' alignment and, during the method optimization step, to set up the “good enough” alignment quality score compensating for small variation between runs in the IM drift times. This ensures that potential conflicting features with drift times close to those “fixed” as references may not still yield positive results. As such the automatic alignment procedure does not introduce artefacts in the analysis process.

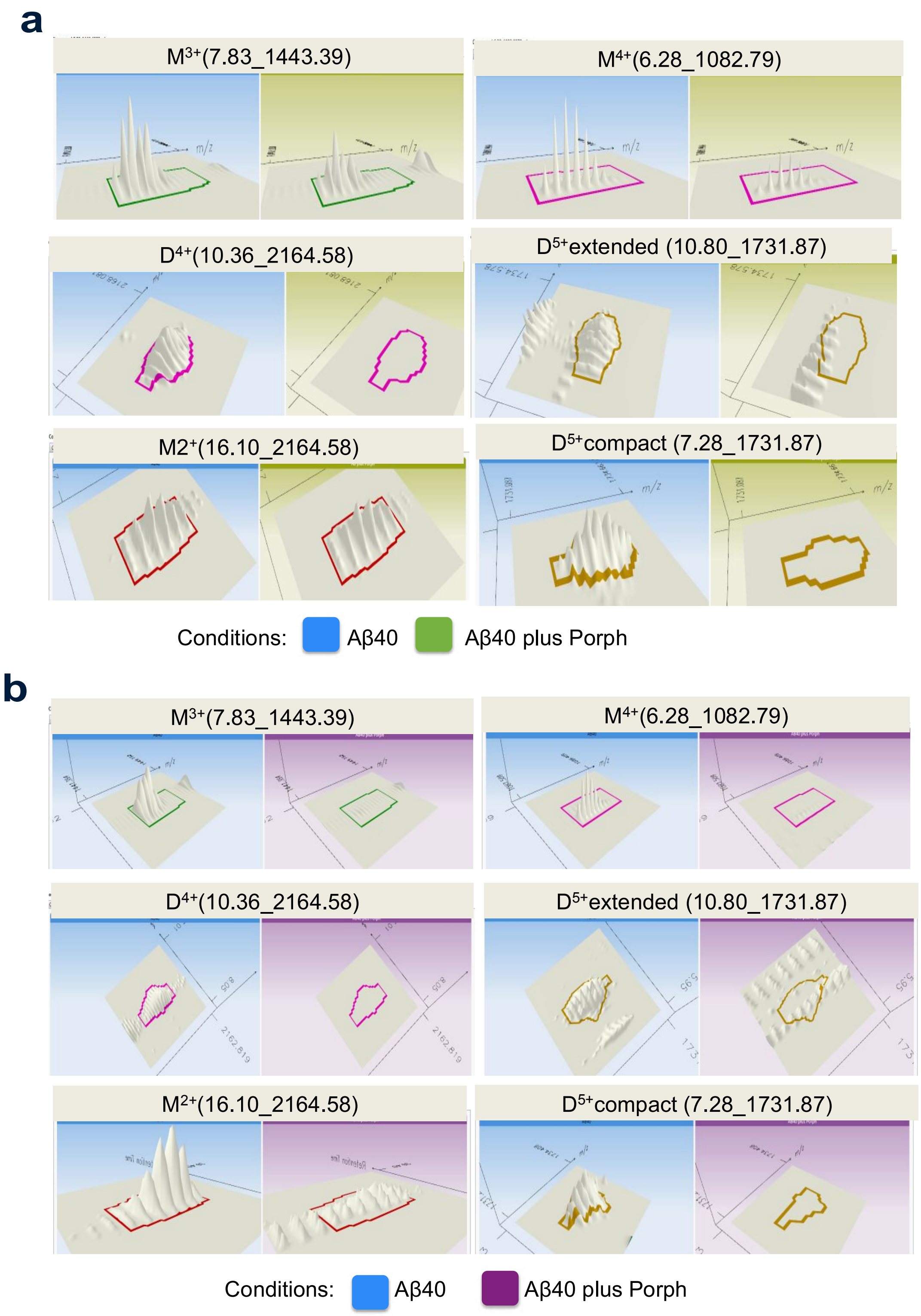

Fig. S6 3D montage *m/z* *vs.* drift time *vs.* intensity measured for a run of the "Aβ40" sample-class and a run of the "Aβ40 plus Porph" sample-class containing Zn-Porph (Aβ40:Zn-Porph, 1:1) at 5 μM (a), at 20 μM (b). On the 3D montage is shown the automated peak picking of the protonated monomers and dimers of Aβ40 assigned to the M^{3+}, M^{4+}, D^{4+}, M^{2+} and to the compact and extended conformations of D^{5+} based on the measured isotopic envelope and drift time (dt)

measurements. To ensure consistent peak picking of the Aβ40 species and matching across all data files, for the peak-picking algorithm only the runs of "Aβ40" sample class were we ticked. However, any run of the "Aβ40 plus Porph" class, which was left un-ticked, was available in the experiment design setup. The end results were an highly reproducible peptide ion-detection and ion-abundance measurement across the "Aβ40" and Aβ40 plus Porph" sample classes

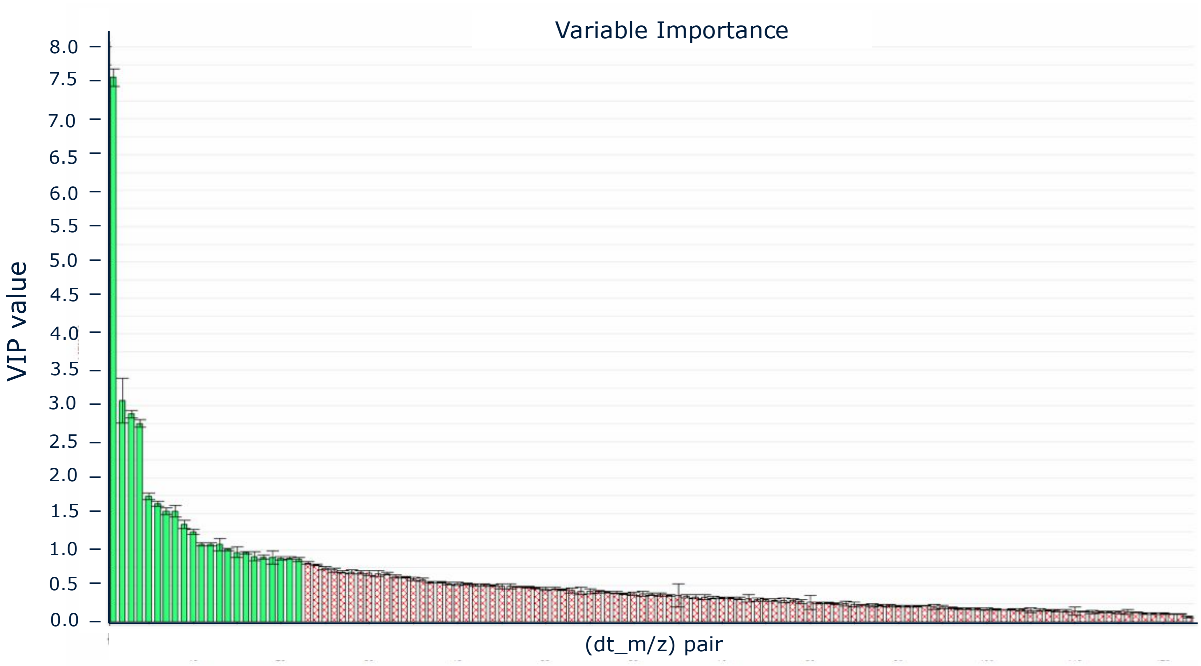

Fig. S7 "Aβ40" sample set at 20 uM. Representative EZinfo VIP plot ranking the *dt_m/z marker pairs* of the "Aβ40" sample-class (shared by the "Aβ40" and "Aβ40 plus Porph" sample-classes or unique of the "Aβ40" class) selected on the S-plot of OPLSA-DA model according the acceptance criteria illustrated on Fig. S7. The bars correspond to the selected features. The height of each bar represents the VIP value as result of the overall contribution of each variable to the model taking into account both p(corr)[1] (variable reliability) and p[1] (variable magnitude) values. Highlighted in green are *dt_m/z marker pairs* with a VIP value higher than one with highest reliability and discriminatory capacity with VIP > 1.0. Some of them correspond to alkali metal adduct commonly observed in ESI-MS

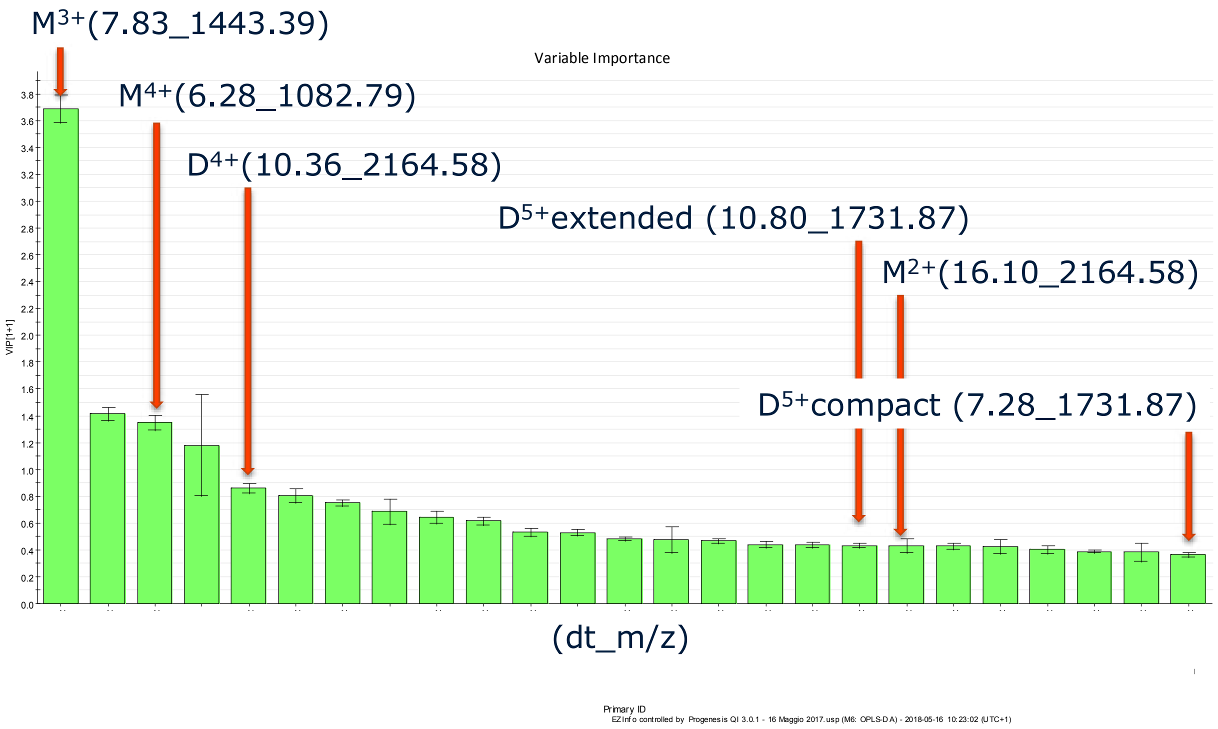

Fig. S8 Aβ40" sample-class at 20 µM: representative EZinfo VIP plot. Zoom of the first region of the VIP plot on Fig. S8 including the most significant *dt_m/z marker pairs* (VIP>1) finally selected to evaluate the inhibitory effect of Zn-Porph on their formation. For clarity, highlighted by arrows are the rt_*m/z* pairs corresponding to the monomeric (M) and dimeric (D) species of Aβ40: M^{3+}(7.83_1443.39), M^{4+}(6.28_1082.79), D^{4+}(10.36_2164.58), extended (10.80_1731.87) and compact (7.28_1731.87) conformations of the D^{5+}. The feature (16.10_2164.58) corresponding to the M^{2+} was selected only in the two sample-sets at 20 µM. The bars not labelled correspond to alkali metal adduct commonly observed in ESI-MS

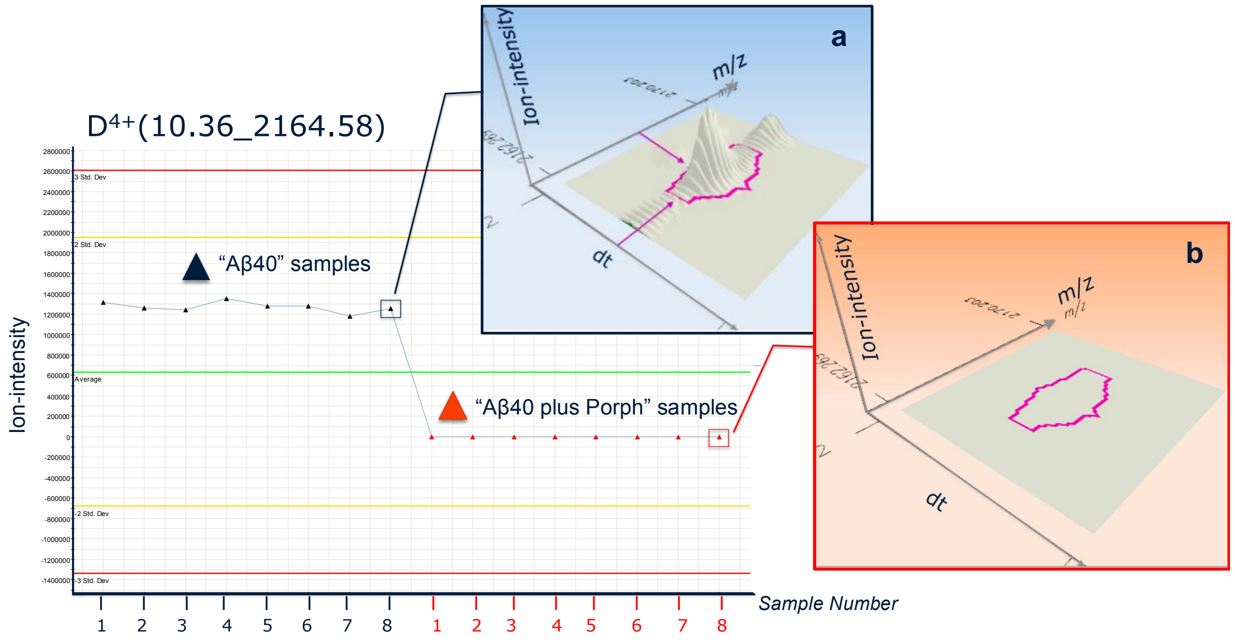

Fig. S9 Representative ion intensity trend plot (XVar trend plot) of the feature corresponding to the D^{4+}(10.36_2164.58) pair in the sample set incubated at 37°C for 2h before injection. On the y-axis is the ion-intensity value measured across the eight samples of the “Aβ40” sample-class (black dots) and the eight samples of the “Aβ40 plus Porph” class (red dots). Number of the sample in the two classes is shown on the x-axis. Ion-intensity values were measured by Progenesis QI software once alignment procedure and the automatic peak picking were performed (Fig.S6). The insets show the zoomed peak picking for the D^{4+}(10.36_2164.58) pair of an “Aβ40” sample (a) and of an “Aβ40 plus Porph” sample (b) based on the measured isotopic envelope and drift time (dt) measurements

Aβ40 species	Primary ID	p[1]	p(corr)[1]	Average ion intensity "Aβ40" class	Std.Dev ion intensity "Aβ40" class	Average ion intensity "Aβ40 plus Porph" class	Std.Dev ion intensity "Aβ40 plus Porph" class
M^{3+}	7.83_1443.39	0.8874	0.9714	4.54e+06	2.18e+05	1.97e+06	4.23e+05
M^{4+}	6.28_1082.79	0.4301	0.9939	8.44e+05	3.98e+04	2.54e+05	3.73e+04
D^{4+}	10.36_2164.58	0.1039	0.9598	3.55e+04	8.68e+03	0	N/A
D^{5+} extended	10.80_1731.87	0.0948	0.9655	3.44e+04	6.58e+03	5.05e+03	4.63e+02
D^{5+} compact	7.28_1731.87	0.0729	0.9840	1.71e+05	2.75e+03	0	N/A
M^{2+}	16.10_2164.58	0.0501	0.7988	3.10e+04	3.51e+04	2.10e+04	4.16e+03

Table S4 Average ion-intensity and standard deviation value of *dt_m/z marker pairs* used to evaluate the inhibitory effect of Zn-Porph (Aβ40:Zn-Porph, 1:1) on their formation in the sample-sets at 5 µM. The *dt_m/z marker pairs* are listed in descending order of their the covariance parameter p[1] value measuring their contribution to the inter-class separation. Due to its high intra-class intensity variability (p(corr)[1] < + 0.9), the dt_*m/z* pair (16.10_2164.58) corresponding to the Aβ40 M^{2+} was automatically excluded from the data matrix of the sample-set at 5 µM

Aβ40 species	Primary ID	p[1]	p(corr)[1]	Average ion intensity "Aβ40" class	Std.Dev ion intensity "Aβ40" class	Average ion intensity "Aβ40 plus Porph" class	Std.Dev ion intensity "Aβ40 plus Porph" class
M^{3+}	7.83_1443.39	0.9206	0.9972	6.71e+06	3.42e+05	4.87e+05	9.36e+04
M^{4+}	6.28_1082.79	0.3523	0.9978	9.74e+05	3.84e+04	6.24e+04	2.30e+04
D^{4+}	10.36_2164.58	0.1265	0.9923	1.18e+05	1.14e+04	0	N/A
D^{5+} extended	10.80_1731.87	0.0740	0.9714	5.40e+04	7.39e+03	1.28e+04	1.47e+03
M^{2+}	16.10_2164.58	0.0634	0.9436	5.05e+04	4.66e+03	1.92e+04	6.81e+03
D^{5+} compact	7.28_1731.87	0.0540	0.9955	2.15e+04	1.60e+03	0	N/A

Table S5 Average ion-intensity and standard deviation value of *dt_m/z marker pairs* used to evaluate the inhibitory effect of Zn-Porph (Aβ40:Zn-Porph, 1:1) on their formation in the sample set at 20 μM. The *dt_m/z marker pairs* are listed in descending order of their the covariance parameter p[1] value measuring their contribution to the inter-class separation

Aβ40 species	Primary ID	p[1]	p(corr)[1]	Average ion intensity "Aβ40" class	Std.Dev ion intensity "Aβ40" class	Average ion intensity "Aβ40 plus Porph" class	Std.Dev ion intensity "Aβ40 plus Porph" class
M^{3+}	7.83_1443.39	0.9037	0.9982	2.75e+07	4.60e+05	2.92e+06	1.14e+06
M^{4+}	6.28_1082.79	0.3278	0.9990	3.50e+06	4.15e+04	2.74e+05	1.06e+05
D^{4+}	10.36_2164.58	0.2057	0.9991	1.27e+06	5.13e+04	0	N/A
M^{2+}	16.10_2164.58	0.1166	0.9598	6.55e+05	2.65e+04	2.30e+05	9.05e+04
D^{5+} extended	10.80_1731.87	0.1108	0.9987	4.83e+05	1.45e+04	1.14e+05	8.01e+03
D^{5+} compact	7.28_1731.87	0.0878	0.9991	2.31e+05	1.01e+04	0	N/A

Table S6 Average ion-intensity and standard deviation value of *dt_m/z marker pairs* used to evaluate the inhibitory effect of Zn-Porph (Aβ40:Zn-Porph, 1:1) on their formation in the sample-sets at 20 μM incubated at 37°C for 2h before injection. The *dt_m/z marker pairs* are listed in descending order of their the covariance parameter p[1] value measuring their contribution to the inter-class separation
